# The clinical impact of glenoid concavity and version on anterior shoulder stability

**DOI:** 10.1016/j.jseint.2024.09.029

**Published:** 2024-11-02

**Authors:** Sebastian Oenning, Clara de Castillo, Elena Jacob, Arne Riegel, Philipp A. Michel, Jens Wermers, Michael J. Raschke, J. Christoph Katthagen

**Affiliations:** a Department of Trauma, Hand and Reconstructive Surgery, University Hospital Muenster, Muenster, Germany; b Department of Orthopaedics, Trauma and Reconstructive Surgery, University Hospital RWTH Aachen, Aachen, Germany; c Department of Radiology, University Hospital Muenster, Muenster, Germany; d Faculty of Engineering Physics, FH Muenster, Muenster, Germany

**Keywords:** Glenohumeral instability, Glenoid concavity, BSSR, Glenoid version, Anterior shoulder instability, Shoulder joint dislocation

## Abstract

**Background:**

In recent biomechanical studies, the importance of glenoid concavity and version for anterior shoulder stability has been highlighted. With this study, we aimed to assess their clinical relevance as stabilizing factors. We hypothesized that low glenoid concavity and low retroversion are associated with anterior glenohumeral instability.

**Methods:**

In this single-center, retrospective case-control study, computed tomography scans of n = 34 patients following acute anteroinferior glenohumeral dislocation between 2015 and 2021 were included. Patients with glenoid fractures and pre-existing glenohumeral pathologies were excluded. In the control group, n = 68 polytrauma patients referred to our level-I-trauma center were included, who showed neither acute nor chronic glenohumeral pathologies. Both groups were matched age- and gender-specifically in a 2:1 ratio. Glenoid concavity was measured according to the bony shoulder stability ratio (BSSR) in anterior-posterior (AP) and superior-inferior (SI) direction. Version was measured by the glenoid vault method.

**Results:**

The instability cohort presented with a lower BSSR (SI) compared to the control group (49.8% vs. 56.9%, *P* = .001). The BSSR (AP) did not differ significantly (30.2% vs. 33.7%, *P* = .163). A higher retroversion was seen in the instability cohort (−13.1° vs. −11.4°; *P* = .041). Subgroup analyses showed higher BSSR (SI) in ≥60-year-old patients compared to ≤30-year-old patients. BSSR (AP) and glenoid version did neither differ age- nor gender-specifically.

**Conclusion:**

Glenoid concavity is a relevant factor for anterior shoulder stability in the clinical setting. In contrast to recent biomechanical studies, glenoid version appears to have only limited clinical impact on anterior stability. Regarding the individual treatment of anterior glenohumeral instability, glenoid concavity should be focused on as an essential bony stabilizing factor.

The understanding of shoulder stability has become more differentiated throughout the last years, and glenoid concavity as well as glenoid version has been investigated in recent studies. Still, the clinical relevance of glenoid concavity and version as bony stabilizing factors in the context of anterior shoulder stability is yet unknown.

The principle of concavity-compression describes the synergy of both the glenoid concavity and the rotator cuff’s compressive force, centering the humeral head within the glenoid, and therefore providing glenohumeral stability.^7^^,^^15^^,^^16^ Regarding glenoid concavity, recent biomechanical studies include finite element analyses, bony glenohumeral models, as well as active-assisted shoulder models, considering the concavity-compression mechanism.^20^^,^^21^^,^^23^^,^^30^ Here it has been shown that the glenoid concavity is an essential factor for anterior shoulder stability, and instability is mainly caused by the loss of concavity.^30^ Highly concave-shaped glenoids were shown to tolerate up to 20% glenoid bone loss, until stability was reduced to the level of native joints with low concavity.^23^ Moroder et al established the computed tomography (CT)-based bony shoulder stability ratio (BSSR), describing the glenoid concavity considering the glenoid radius and depth.^21^ However, the role of the glenoid concavity in a clinical setting and physiological ranges of concavity are yet unknown.

Regarding the glenoid version, a mild retroversion can be observed in physiological conditions. In the following, 0° equals a neutral glenoid version, while positive and negative numeric values describe anteversion and retroversion, respectively. The most common methods of measuring glenoid version include the Friedman method and the glenoid vault method. According to Friedman et al, a line is drawn from the most medial point of the scapula to the center of a line connecting the anterior and posterior glenoid rims.^4^ According to Matsumura et al, describing the glenoid vault method, a line connecting the tip of the triangular-shaped glenoid vault and the center of the glenoid articular surface is used for measuring glenoid version. Thus, the individual morphology of the scapular body cannot confound the measurement.^17^ For the glenoid vault method, physiological ranges of −8.9° (±2.7°)^17^ and −8° (±4.9°)^2^ retroversion are described in literature. Regarding the Friedman method, smaller numeric values are described with a physiological range of −2.1° (±4.7°).^2^

Biomechanically, Eichinger et al showed that the glenoid version influences both anterior and posterior shoulder stability in a linear correlation. In their model, every 1° increase of anteversion led to a 6% decrease of anteroinferior dislocation force.^3^ Also, as Imhoff et al pointed out, the humeral head position is influenced by glenoid version. With every 5° increase of retroversion, the humeral head was positioned about 2 mm more posteriorly within the glenoid cavity.^9^

Clinically, a correlation between increased retroversion and posterior shoulder instability is described in several studies.^5^^,^^6^^,^^11^^,^^12^^,^^18^^,^^24^^,^^26^^,^^28^ Regarding anterior shoulder stability, however, the clinical impact of glenoid version is not as clear. Few studies have yet analyzed patient cohorts with anterior instability. In some of these studies, anterior instability collectives were associated with a slightly decreased retroversion compared to control cohorts without shoulder instability.^1^^,^^8^ However, glenoid version in the instability groups did not clearly exceed physiological ranges. Furthermore, Privitera et al found no significant difference in glenoid version between asymptomatic and anteriorly unstable patients.^28^ Thus, the clinical relevance of glenoid version in the context of anterior shoulder stability yet remains unclear.

In this study, the clinical impact of glenoid concavity and glenoid version on anterior shoulder stability was analyzed. Comparing patients with anterior instability to a control cohort without shoulder instability, we hypothesized that anterior instability is associated with lower glenoid concavity and less glenoid retroversion.

## Materials and methods

### Study design

**Figure 1:**
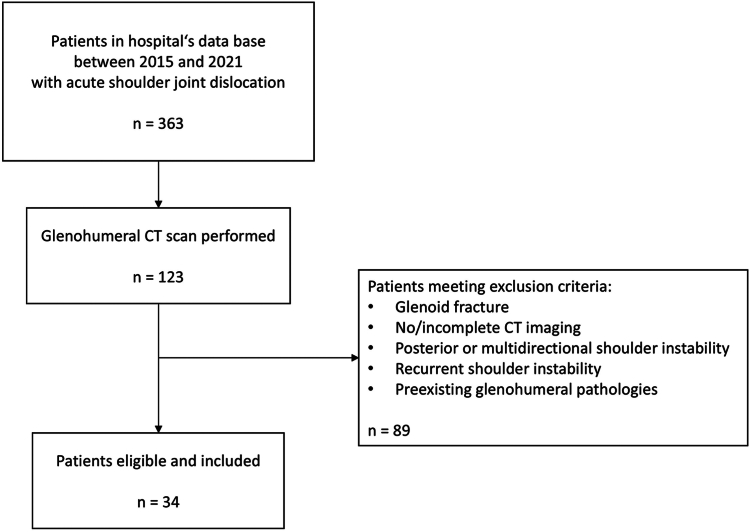
Flow diagram showing the number of patients eligible and included in this study after applying inclusion and exclusion criteria. *CT*, computed tomography.

This retrospective case-control study was performed at the Department of Trauma, Hand and Reconstructive Surgery of the University Hospital Münster, Germany, a level-1-trauma center, and approved by the institutional review board (IRB No. 2021-607-f-S, University of Münster, Germany). CT scans of patients presenting with an acute, anteroinferior shoulder joint dislocation between 2015 and 2021 were evaluated and compared to a control cohort. Exclusion criteria included glenoid fractures, incomplete glenoid imaging in the CT scan, previous episodes of shoulder joint dislocation, multidirectional shoulder instability, and preexisting shoulder joint pathologies. Patient data were obtained via the hospital’s documentation system, Orbis (Dedalus, Bonn, Germany), and n = 34 patients were included in the instability cohort. Detailed numbers of patients after applying inclusion and exclusion criteria are presented in Figure 1.

The control cohort was comprised of polytraumatized patients admitted to our hospital from January 2020 to October 2021, receiving polytrauma CT scans, including glenohumeral joint imaging. Patients without acute and chronic glenohumeral pathologies were included in the control collective. Gender- and age-dependent matching in a 2:1 ratio was performed between the instability and control cohorts, resulting in n = 68 patients within the control group. Specifically, for every patient within the instability cohort, two same-gender patients were matched, in which the equal-sided shoulder was analyzed. Age-specific matching was performed as precise as possible. For n = 20 instability patients, two control patients of ±2 years of age were matched. The maximum age difference within the remaining matched patients was six years.

In addition, subgroups were formed in order to examine the influence of gender (female vs. male) and age (≤30 vs. ≥60 years of age) on glenoid concavity and version.

### Measurements

Radiological measurements were acquired with Aquarius iNtuition (version 4.4, TeraRecon, Durham, NC, USA) using individual multiplanar reconstruction of the CT scan data. CT scan thickness was 1-1.5mm.

Joint-specific coordinate systems were established by creating superiorinferior (SI) and anterior-posterior (AP) axes aligned to the most superior, inferior, anterior, and posterior points of the glenoid rim, respectively. The mediolateral axis was added orthogonally to both, the SI and AP axes.

Glenoid concavity was measured according to the CT-based BSSR, including glenoid radius (r) and depth (d).^21^

$$BSSR=\frac{1-{\left(\frac{r-d}{r}\right)}^{2}}{\frac{r-d}{r}}$$

**Figure 2:**
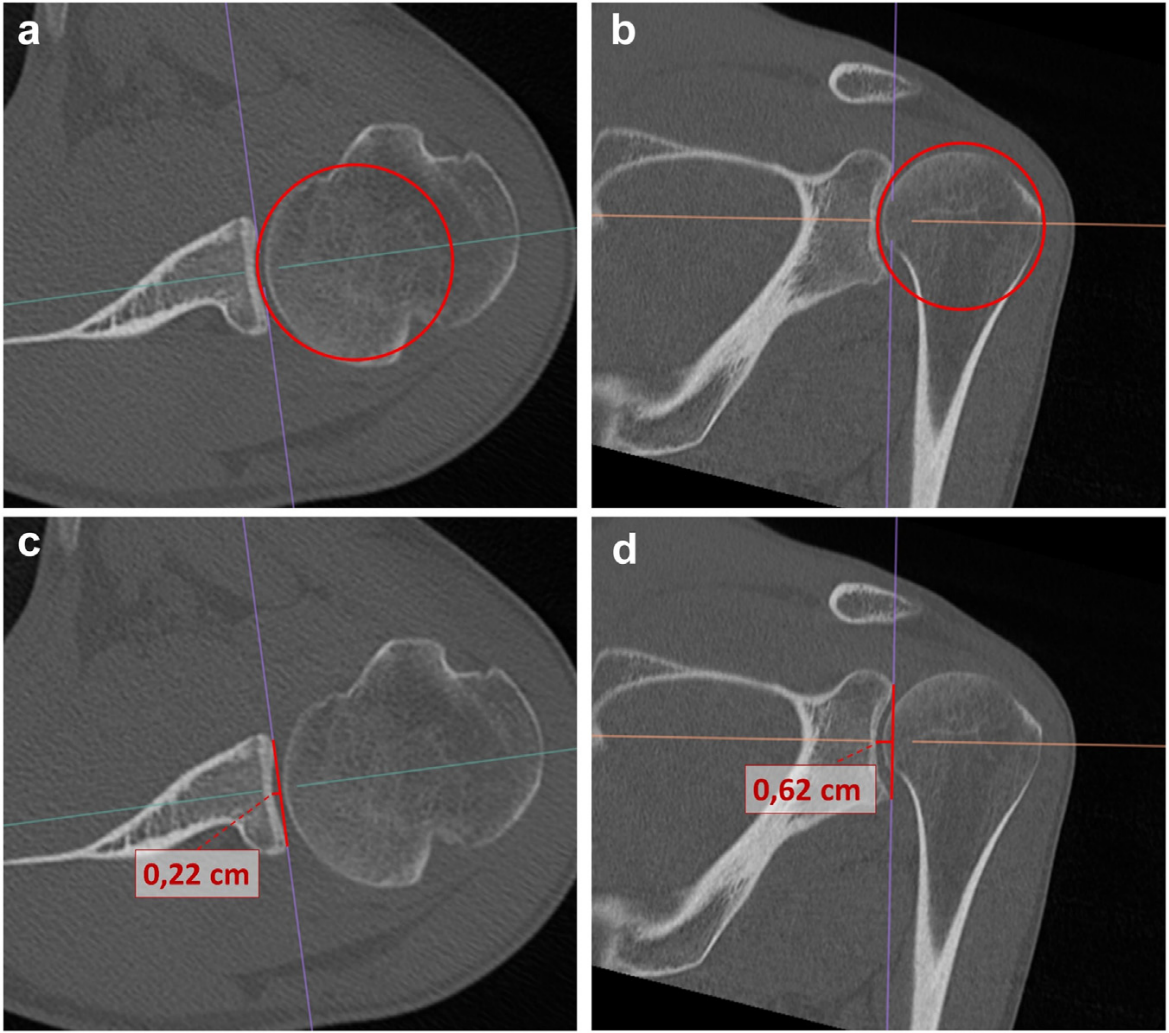
Measurement of the glenoid radius with the best-fit-circle method (**a**, **b**) and the glenoid depth (**c**, **d**). Both were measured in axial (**a**, **c**) and coronal (**b**, **d**) planes.

Measurements of the BSSRs were performed in both the coronal and axial planes, so that the SI concavity (BSSR (SI)) and AP concavity (BSSR (AP)) were analyzed, respectively. The glenoid radius was measured using the best-fit-circle method as described by Kuberakani et al^14^ (see Fig. 2). The glenoid depth (d) was measured by defining the widest SI and AP glenoid diameters, which equal the SI and AP axes, respectively. Orthogonally to each axis, the maximum glenoid depth was measured at each axis’ center point in both the coronal and axial planes (see Fig. 2).

**Figure 3:**
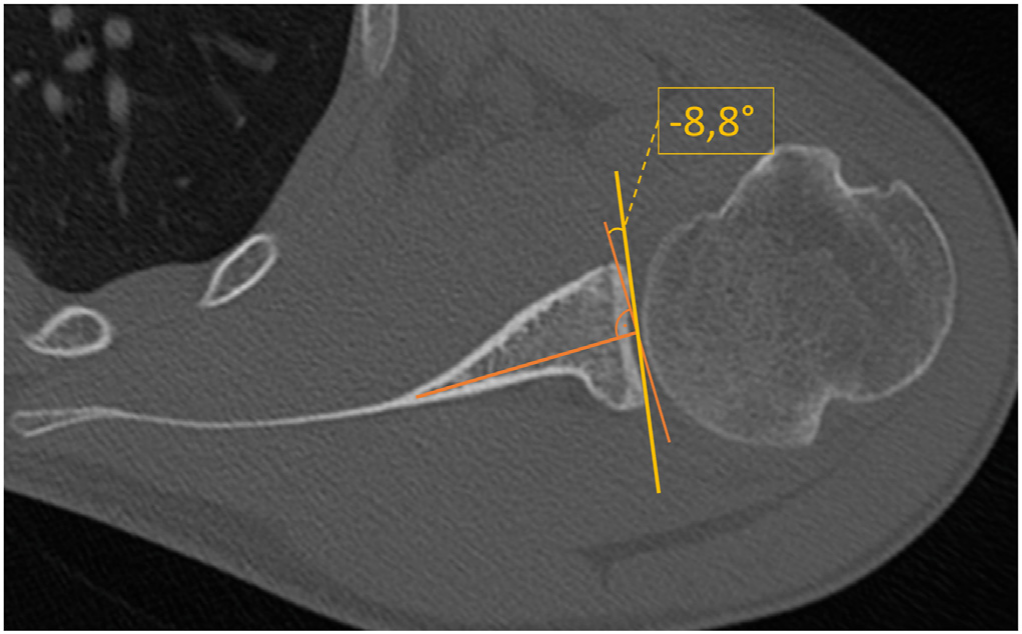
Measurement of glenoid version based on the glenoid vault method, as described by Matsumura et al.

The measurement of glenoid version was based on the glenoid vault method as described by Matsumura et al.^17^ While Matsumura et al aligned the planes of CT scans to the individual scapula body, in this study, the previously described coordinate system aligned to the glenoid was used. Except different CT plane alignments, the measurement of glenoid version was performed analogously to Matsumura et al. In an axial plane, a line was then drawn connecting the tip of the triangle-shaped glenoid vault and the center of the glenoid articular surface. A perpendicular line indicated 0° of glenoid version and was then used for measuring the individual, patient-specific version (see Fig. 3).

### Statistical analysis

An *a priori* power analysis using G∗Power (version 3.1.9.7; Heinrich Heine Universität, Düsseldorf, Germany) was performed to determine the necessary sample size. Here, the mean BSSR (AP) values of the cadaveric study of Wermers et al were used and compared to five BSSR (AP) test measurements within the instability cohort of this study. With an alpha level of 0.05, a power of 0.95, and an effect size of d = 0.942, the required numbers of patients for unpaired t-tests were calculated. Here, numbers of n = 23 patients for the instability cohort and n = 45 patients for the control group were defined.

Statistics were performed using GraphPad Prism (GraphPad Software Inc., San Diego, CA, USA). Descriptive statistics, including median and mean values, standard deviation, range, as well as 25^th^ and 75^th^ percentiles, were calculated for all variables. Normal distribution was assessed graphically via a quantile-quantile plot (Q-Q plot) as well as the Shapiro-Wilk test.

A level of *P* < .05 was deemed significant. For group comparisons, the t-test was used. For parameters not showing normal distribution, the Mann-Whitney U test was applied, additionally. This was performed for both, comparing the instability and control cohorts, as well as analyzing age- and gender-specific subgroups. Correlation between different parameters was tested using a linear regression model. Binary logistic regression was used to analyze the impact of concavity on the occurrence of shoulder instability, presented with odds ratios (ORs) and the OR’s 95% confidence intervals (CIs). A post-hoc power analysis was performed to verify the preliminarily set CI of 95%.

## Results

### Study population

In the instability cohort, n = 34 patients were included, while the control cohort consisted of n = 68 patients after matching. Throughout all included patients, the mean age was 48 years (±19.9; 18-92). Within instability and control cohorts, the patients’ mean age was 46.9 (±20.3) and 48.6 (±19.9) years, respectively. Within each group, 26.5% of patients were female, 73.5% were male. Within the instability cohort, n = 19 patients presented with a right shoulder injury, while in n = 15 patients the left shoulder was affected. Regarding the mechanism of injury within the instability cohort, two patients presented with atraumatic shoulder dislocations and hyperlaxity. Five patients sustained dislocations after seizures, while the remaining n = 27 patients described adequate trauma (falling, sports injuries, vehicle/traffic accidents). A spontaneous glenohumeral reposition was observed in three patients, while n = 31 patients required closed reduction.

### Primary outcome

**Figure 4:**
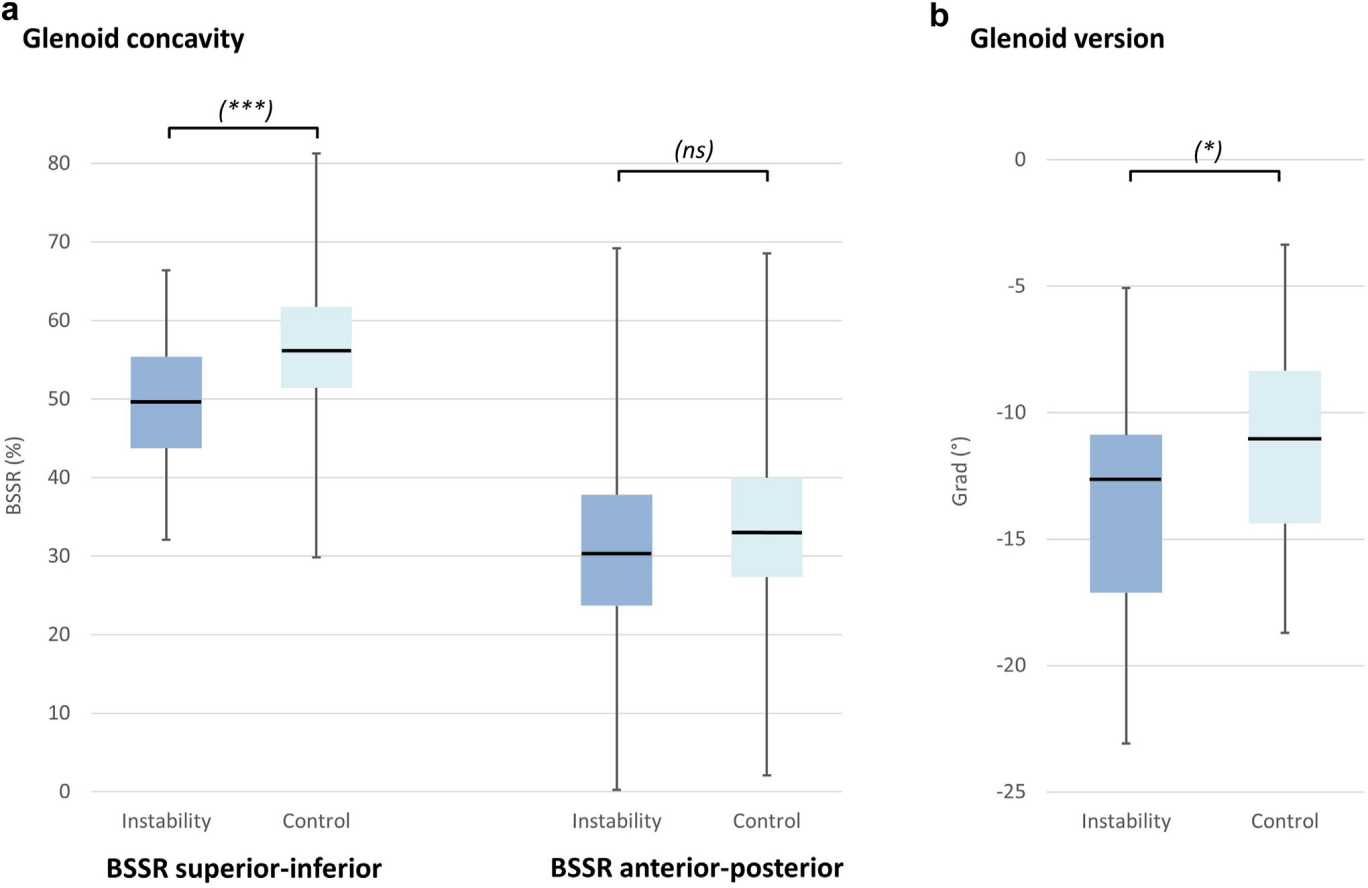
Boxplots and analyses of glenoid concavity (**a**) and version (**b**) between instability cohort (*dark blue*) and control cohort (*light blue*). *BSSR*, bony shoulder stability ratio.

Glenoid concavity and version were measured and compared between the instability and control cohort (see Fig. 4). The mean BSSR (SI) in the instability group was 49.8% (±9.0), while the control cohort showed a mean BSSR (SI) of 56.9% (±9.9). Therefore, patients in the instability cohort presented with a significantly lower concavity in the SI axis compared to the control group (*P* = .0007). Regarding the glenoid concavity in the AP axis, the difference between instability and control groups showed no statistical significance (*P* = .1634). Here, the instability group showed a mean BSSR (AP) of 30.15% (±13.63), while the control group presented with a BSSR (AP) of 33.72% (±11.44). Details are described in Table I.

**Table I:** Detailed values (mean, standard error of mean, range) and statistical analyses of glenoid radius, depth, the bony shoulder stability ratio (BSSR) and glenoid version.

	Instability cohort	Control cohort	*P* value
Glenoid radius (coronal)	36.54 (±7.8; 22.8-64)	32.59 (±4,91; 23,4-50.5)	**.0048**^(b)^
Glenoid radius (axial)	43.72 (±14.81; 19.25-81)	36.58 (±8.2; 22.65-61)	**.0241**^(b)^
Glenoid depth (coronal)	3.72 (±0,79; 1.9-5.36)	4.18 (±0.88; 2.03-6.2)	**.0115**^(a)^
Glenoid depth (axial)	1.73 (±0.85; 0-3.42)	1.89 (±0.89; 0.01-4.95)	.5974^(b)^
BSSR (SI/coronal)	49.82 (±9.09; 31.95-66.42)	56.93 (±9.94; 29.62-81.28)	**.0007**^(a)^
BSSR (AP/axial)	30.15 (±13.63; 0-69.19)	33.72 (±11.44; 1.81-68.57)	.1634^(b)^
Glenoid version	−13.14 (±4.38; −22.6 to −5)	−11.44 (±3.66; −18.7 to −3.3)	**.0407**^(a)^

Radius, depth and BSSR were assessed in coronal planes in a superior-inferior (SI) axis, as well as in axial planes in an anterior-posterior (AP.) axis. Statistical analysis was either performed by t-tests ^(a)^ or Mann-Whitney U tests ^(b)^ depending on the distribution of values. Significant values (*P* < .05) are shown in bold.

*BSSR*, bony shoulder stability ratio.

Binary logistic regression analyses showed that with every 1% increase in BSSR (SI), the risk of anteroinferior shoulder instability decreases by 8% (OR 0.92; 95% CIs 0.87-0.97; *P* = .0017). Regarding a 1% increase of the BSSR (AP), the decreased risk of shoulder instability was not significant (OR 0.98; 95% CIs 0.94-1.01; *P* = .1695).

In the overall study population, BSSR (SI) and BSSR (AP) showed a low correlation with a determination coefficient of R^2^ = 0.23 in a linear regression model.

For evaluation of the BSSR, glenoid radius and depth were assessed in both axial and coronal planes. Radius and depth were analyzed separately to detect specific differences masked by the BSSR. Here, the instability group presented with a significantly higher glenoid radius in both planes. Regarding glenoid depth in coronal planes, significantly higher values were seen in the control group compared to patients in the instability cohort (*P* = .0115), while in axial planes, the glenoid depth did not differ significantly (*P* = .5974). These results are consistent with the BSSR providing significant differences only in coronal planes. Details are shown in Table I.

Assessment of glenoid version showed more retroversion in the instability cohort with a mean glenoid version angle of −13.14° (±4.38; −22.6 to −5). In the control group, a mean glenoid version of −11.44° (±3.66; −18.7 to −3.3) was seen, showing significantly less retroversion (*P* = .0407). In the linear regression model, the glenoid version did not correlate with BSSR (SI) and BSSR (AP) with determination coefficients of R^2^ = 0.0144 and R^2^ = 0.0016, respectively.

### Subgroup analyses

**Figure 5:**
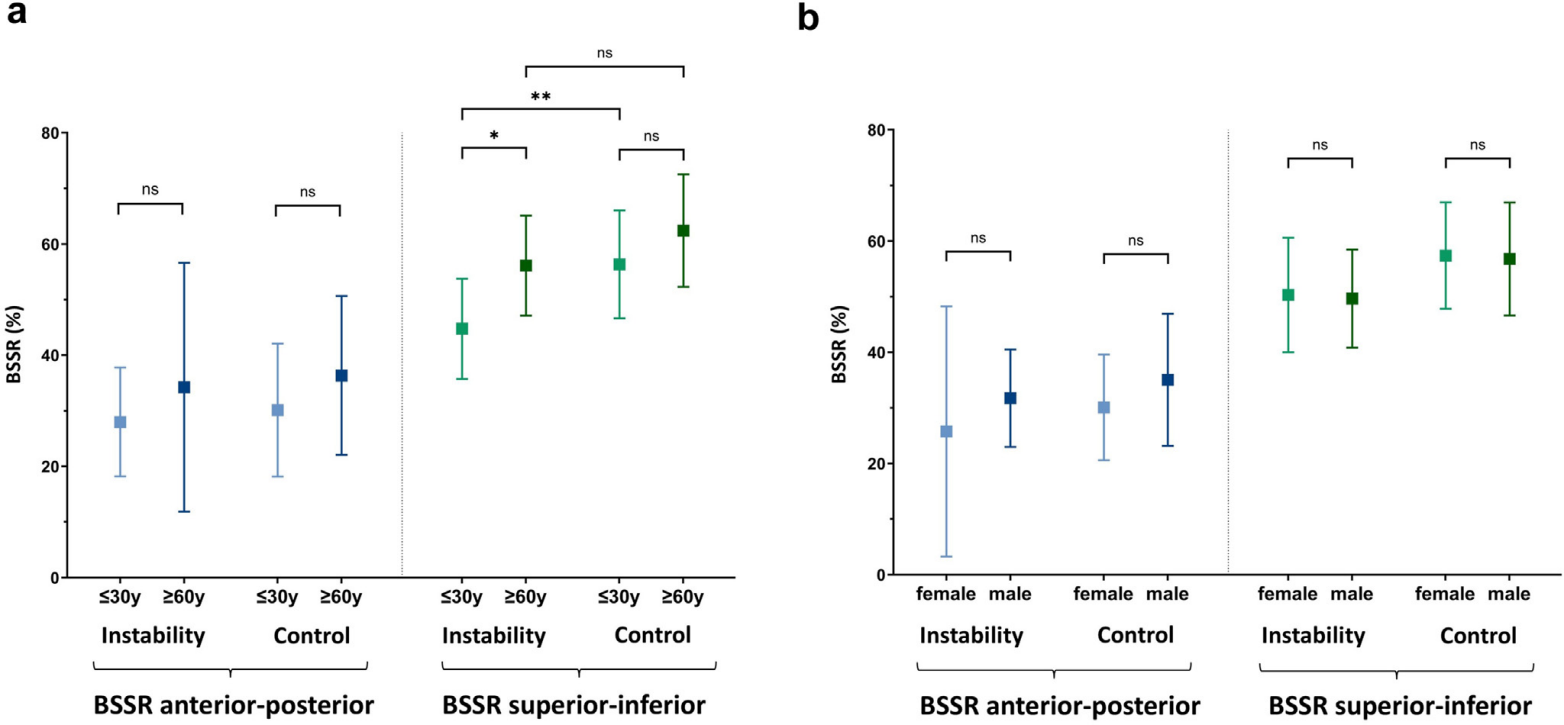
Age-specific (**a**) and gender-specific (**b**) subgroup analyses of the glenoid concavity in axial planes (AP, *blue*) and coronal planes (SI, *green*) are shown. Patients were compared regarding (**a**) age (≤30 years vs. ≥60 years) and (**b**) gender (female vs. male). *BSSR*, bony shoulder stability ratio.

**Figure 6:**
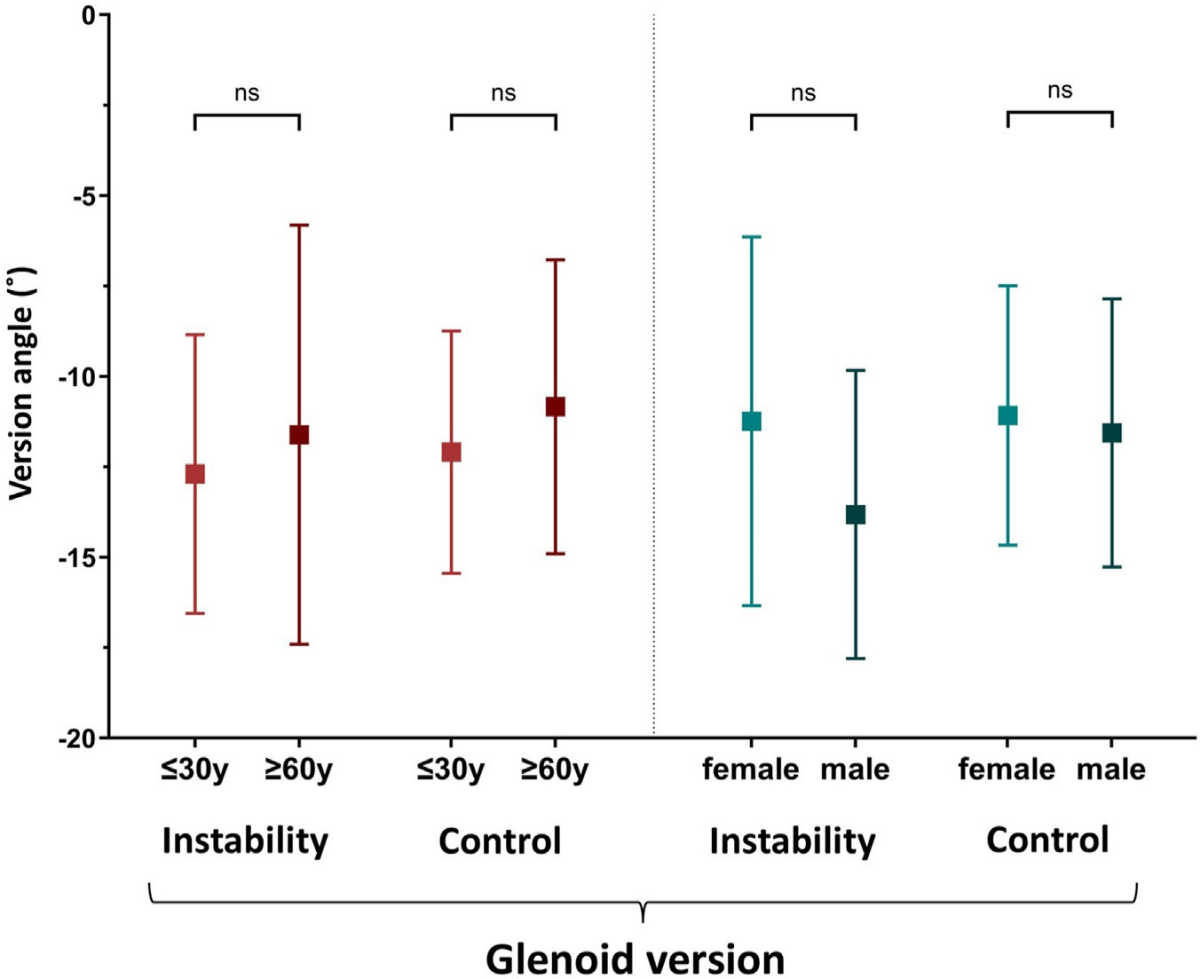
Age-specific and gender-specific subgroup analyses of the glenoid version. Patients were compared regarding age (≤30 years vs. ≥60 years) and gender (female vs. male).

Within the instability and control cohorts, age- and gender-specific subgroups were defined (see Figs. 5, *A* and B, 6).

To evaluate age-dependent differences in concavity and version and their impact on shoulder stability, patients ≤30 years of age were compared to patients ≥60 years of age (see Fig. 5, *A*). Regarding the BSSR (AP), no significant differences were seen between ≤30 and ≥60-year-old patients within both the instability and control cohorts (*P* = .4409; *P* = .19, respectively). In contrast, ≤30-year-old patients showed a lower mean BSSR (SI) than ≥60-year-old patients within the instability cohort (44.75% (±9.02) vs. 56.11% (±8.98), *P* = .0218). While a significantly lower BSSR (SI) in the instability cohort compared to the control group was evident in ≤30-year-old patients (44.75% (±9.02) vs. 56.33% (±9.73), *P* = .0064), in ≥60-year-old patients, no difference was seen between the instability and control cohorts (56.11% (±8.98) vs. 62.4% (±10.12), *P* = .1647). Within the control group, BSSR (SI) differences between ≤30 and ≥60-year-old patients were not significant (*P* = .091). Regarding glenoid version, no age-specific differences were found (*P* ≥.347) (see Fig. 6).

Comparing female to male patients within each cohort, no significant differences were seen regarding BSSR (AP) (*P* ≥.1157) and BSSR (SI) (*P* ≥.8273) (see Fig. 5, *B*). Also, glenoid version did not show gender-dependent differences within the instability cohort (female = −11.24° (±5.1) vs. male = −13.82° (±3.99), *P* = .1326) and control cohort (female = −11.08° (±3.59) vs. male = −11.56° (±3.51), *P* = .6321).

## Discussion

In this study evaluating the clinical relevance of glenoid concavity and version for anterior shoulder instability, we can summarize the following main findings: (1) Anterior shoulder instability is associated with a lower glenoid concavity in coronal planes. In axial planes, the same tendencies were seen; however, without showing statistical significance. (2) The role of glenoid version in the context of anterior glenohumeral stability remains controversial, since in this study a higher retroversion was seen in the instability cohort compared to the control group.

Regarding glenoid concavity, the results of this study fall in line with previous biomechanical studies. Moroder et al described the stabilizing effect of glenoid concavity through finite element analysis. In cases of osseous Bankart lesions, they suggested that the loss of concavity might be a more precise parameter indicating anterior glenohumeral instability than conventional, two-dimensional methods measuring the glenoid defect size.^20^^,^^21^ Previous biomechanical results from our working group confirmed the importance of glenoid concavity. In an osteochondral model using cadaveric glenoids and humeral heads, a high correlation between concavity and stability was found, while the loss of concavity served as a precise predictor for anterior shoulder instability.^30^ This was confirmed in an active-assisted cadaveric model including soft tissue and the rotator cuff’s compressive forces, resembling the physiological, stabilizing mechanism of concavity compression.^16^^,^^23^

This study underlines the importance of glenoid concavity in a clinical setting. SI concavity was lower in the instability cohort. The same tendency of lower AP concavity was found in the instability group, and the difference might become significant with a larger study population. One could also suggest that inferior glenoid concavity plays a more important role in preventing anteroinferior glenohumeral dislocation than anterior concavity. This could be explained by other, mainly anteriorly located, anatomical structures, such as the coracoid or the conjoint tendons, as well as individual labral morphology and anterior capsular tension, helping to prevent anterior humeral head translation. However, further biomechanical studies are required to draw final conclusions to this assumption. The specific anteroinferior concavity within the track of humeral head dislocation, as well as the correlation of glenoid concavity, version, and inclination should be included.

Regarding glenoid version in the context of anterior shoulder stability, the results in this study do not support the findings of previous biomechanical and clinical studies. Biomechanically, Eichinger et al found a linear correlation between glenoid version and both anterior and posterior stability, with increased anteversion causing anterior instability and vice versa.^3^ Imhoff et al described a more posterior humeral head position in case of increased glenoid retroversion, leading to increased posterior instability.^9^ While the association of increased glenoid retroversion and posterior glenohumeral instability became apparent in several clinical studies,^5^^,^^6^^,^^12^^,^^24^^,^^26^^,^^28^ the clinical correlation of glenoid anteversion and anterior stability remains ambiguous. Only a few studies compared a cohort with anterior instability to patients without shoulder instability. Privitera et al did not find a significant difference in glenoid version between both groups.^28^ Hohmann et al and Aygün et al describe a slightly higher anteversion in anterior instability cohorts; however, the amount of glenoid version barely exceeds physiological ranges.^2^^,^^17^ In this study, the anterior instability cohort controversially presented with higher retroversion compared to the control cohort, leading to the assumption that glenoid version provides only limited influence on anterior glenohumeral stability.

Another possible explanation for the presented results could be a reciprocal, anatomical adaption of glenoid version and concavity. For example, an increased glenoid retroversion would counteract a low native concavity, reducing anterior instability. However, the low correlation between glenoid concavity and version in this study does not support this theory. Still, we consider the relation between concavity and version to be worth analyzing in larger, differentiated patient cohorts to produce more detailed results.

In subgroup analyses, it was evident that, especially in coronal planes, older patients presented with a higher glenoid concavity. We consider this finding to be mainly caused by degenerative changes leading to increased central glenoid depth, and therefore increased concavity.^10^^,^^29^ It was striking that in ≥60-year-old patients, axial and coronal glenoid concavity did not differ between the instability and control cohorts. This leads to the suggestion that concavity only plays a minor role in older patients, while low concavity in young patients was evidently associated with anterior shoulder instability. Regarding gender-specific analyses, no relevant differences were seen. Glenoid version did neither show age- nor gender-specific differences.

Limitations of this study include its retrospective study design. A higher number of patients included in this study would have been desirable; however, the required study population according to the power analysis was exceeded. The higher mean age of the instability cohort compared to larger shoulder dislocation cohorts^13^^,^^22^^,^^25^ must be mentioned as well. A reason for this could be the fact that younger patients were shown to have a higher risk of sustaining Bankart fractures,^19^ making them not eligible for inclusion. Also, a relevant number of especially younger patients suffering from anteroinferior shoulder dislocation without glenoid bone loss could not be included due to reasonable diagnostic algorithms. Especially in younger patients, magnetic resonance imaging is preferred over CT scans in the absence of signs of bony glenoid injury in the initial X-ray images.

Another factor limiting the accuracy of the results is the comparably high slice thickness of 1-1.5 mm of the polytrauma CT scans, which were performed in an emergency setting and retrospectively used to generate the control cohort. Minimal changes in concavity and version can significantly alter the measurements, and therefore thinner CT scan slices would have increased the validity of this study. Also, possible degenerative, cartilage lesions, especially in older patients, as well as individual labral morphology, were not detected by CT scans.

The method of measuring glenoid version has to be mentioned as a possible limitation, as well. The most commonly used techniques include the Friedman method as well as glenoid vault methods.^4^^,^^17^^,^^27^ Since the polytrauma CT scans of the control cohort do not regularly include the most medial aspects of the scapular body, the Friedman method could not be applied in this study. Also, the glenoid vault method according to Matsumura had to be adjusted, as the entire scapula body is necessary for CT plane alignment.^17^ In this study, the coordinate system aligned to the glenoid surface in order to measure concavity was also used for assessing glenoid version. This slight CT plane deviation might explain that previously published ranges of retroversion are exceeded in both the instability and control cohorts of this study. Still, we assume that the comparison of version between both cohorts and the correlation between version and concavity within this study remains reliable.

Future studies including a higher number of patients and high-quality CT imaging should be performed to confirm the clinical relevance of glenoid concavity in both native joints and in the presence of glenoid bone loss. Also, the yet controversial role of glenoid version in the context of anterior shoulder stability should be addressed. Furthermore, magnetic resonance imaging scan evaluation could include the morphology of glenoid cartilage and labrum, resulting in individual glenolabral concavity and version.

## Conclusion

Glenoid concavity is a relevant factor for anterior shoulder stability, not only in biomechanical models but also in a clinical setting.

The role of glenoid version remains controversial, since in this study it appears to have only limited clinical impact on anterior stability.

In an individualized therapeutic approach to anterior glenohumeral instability, glenoid concavity should be focused as an essential bony stabilizing factor.

## Disclaimers

Funding: The Open Access Publication Fee was paid by the Open Access Publication Fund of the University of Münster. There was no additional external funding received for this study. The funders had no role in study design, data collection and analysis, decision to publish, or preparation of the manuscript.

Conflict of interest: The authors, their immediate families, and any research foundations with which they are affiliated have not received any financial payments or other benefits from any commercial entity related to the subject of this article.
